# Comparison of high-dose IVIG and rituximab versus rituximab as a preemptive therapy for de novo donor-specific antibodies in kidney transplant patients

**DOI:** 10.1038/s41598-023-34804-6

**Published:** 2023-05-11

**Authors:** Hyung Woo Kim, Juhan Lee, Seok-Jae Heo, Beom Seok Kim, Kyu Ha Huh, Jaeseok Yang

**Affiliations:** 1 Department of Internal Medicine, Yonsei University College of Medicine, Seoul, Korea; 2 Department of Surgery, Yonsei University College of Medicine, Seoul, Korea; 3 Division of Biostatistics, Department of Biomedical Systems Informatics, Yonsei University College of Medicine, Seoul, Korea

**Keywords:** Kidney, Renal replacement therapy, Transplant immunology

## Abstract

De novo donor-specific antibody (dnDSA) is associated with a higher risk of kidney graft failure. However, it is unknown whether preemptive treatment of subclinical dnDSA is beneficial. Here, we assessed the efficacy of high-dose intravenous immunoglobulin (IVIG) and rituximab combination therapy for subclinical dnDSA. An open-label randomized controlled clinical trial was conducted at two Korean institutions. Adult (aged ≥ 19 years) kidney transplant patients with subclinical class II dnDSA (mean fluorescence intensity ≥ 1000) were enrolled. Eligible participants were randomly assigned to receive rituximab or rituximab with IVIG at a 1:1 ratio. The primary endpoint was the change in dnDSA titer at 3 and 12 months after treatment. A total of 46 patients (24 for rituximab and 22 for rituximab with IVIG) were included in the analysis. The mean baseline estimated glomerular filtration rate was 66.7 ± 16.3 mL/min/1.73 m^2^. The titer decline of immune-dominant dnDSA at 12 months in both the preemptive groups was significant. However, there was no difference between the two groups at 12 months. Either kidney allograft function or proteinuria did not differ between the two groups. No antibody-mediated rejection occurred in either group. Preemptive treatment with high-dose IVIG combined with rituximab did not show a better dnDSA reduction compared with rituximab alone.

**Trial registration: **IVIG/Rituximab versus Rituximab in Kidney Transplant With de Novo Donor-specific Antibodies (ClinicalTrials.gov Identifier: NCT04033276, first trial registration (26/07/2019).

## Introduction

The presence of donor-specific antibody (DSA) and antibody-mediated rejections are poor prognostic factors for graft survival and a major cause of graft failure in kidney transplant (KT) patients^1,2^. There is increasing evidence that reducing DSA levels is associated with better long-term kidney allograft survival in patients with antibody-mediated rejection. Monitoring for DSA after kidney transplantation has been widely used.

The allograft survival rate was poorer in patients with de novo (dnDSA) than in those with preexisting DSA, although antibody-mediated rejection can occur in both patients with preexisting DSA and dnDSA^3^. dnDSA led to subclinical antibody-mediated rejection in 50% of patients within 6 months and was accompanied by clinical acute antibody-mediated rejection in 52.9% and chronic antibody-mediated rejection in 38.2% within 1 year^4,5^. Subclinical antibody-mediated rejection with dnDSA results in graft loss in 50% of patients in 8 years, and clinical antibody-mediated rejection with dnDSA results in graft loss in 50% of patients in 3 years^3,4^. In particular, early dnDSA development within 1 year after transplantation was associated with lower allograft survival than late dnDSA development^6^. Although many patients with dnDSA have stable kidney function without specific abnormalities on kidney biopsies, dnDSA was associated with poor prognosis for kidney allografts regardless of rejection^7,8^.

Therefore, it is important to actively control dnDSA and accompanying antibody-mediated rejection to improve kidney allograft outcomes. In previous studies, dnDSA reduction was identified only after intravenous immunoglobulin (IVIG) or rituximab use during desensitization^9,10^ or rejection treatment^11^. However, treatment outcomes for dnDSA and antibody-mediated rejection are poor, especially chronic antibody-mediated rejection with dnDSA^3,4,12,13^. Interestingly, treating subclinical antibody-mediated rejection with DSA, mainly dnDSA, using plasmapheresis, IVIG, and rituximab yielded better outcomes than untreated subclinical rejection and treated clinical antibody-mediated rejection, suggesting the importance of early diagnosis and treatment^14^. However, no randomized clinical trial has evaluated the effectiveness of preemptive treatment for subclinical dnDSA in patients undergoing KT. This study compared preemptive combination therapy with high-dose IVIG and rituximab with rituximab alone for reduction effects of subclinical dnDSA.

## Methods

### Trial design

This study was an open-label, randomized, controlled clinical trial conducted at two large tertiary academic medical centers (Severance Hospital and Seoul National University Hospital) in Korea between January 2019 and October 2021. This study was approved by the Institutional Review Board (IRB) of Yonsei University Health System (IRB No. 4–2018-0359) and Seoul National University Hospital (IRB No. H-1712–158-912). The protocol was registered on ClinicalTrials.gov (Identifier: NCT04033276, first trial registration 26/07/2019) and conducted in accordance with the principles of the Declaration of Istanbul and the Declaration of Helsinki. All patients provided informed consent. The requirement for informed consent of patients who were retrospectively recruited from Severance Hospital was waived by IRB of Yonsei University Health System (IRB No. 4–2022-1125) because of the retrospective nature of the study.

### Participants, randomization, and intervention

KT patients with subclinical class II dnDSA were screened. Participants who met the following criteria were included: (i) age ≥ 19 years; (ii) estimated glomerular filtration rate (eGFR) > 20 ml/min/1.73 m^2^; and (iii) mean fluorescent intensity (MFI) ≥ 1000 of the DR or DQ DSA. Exclusion criteria were age < 19 years, multi-organ transplant, active malignant disease in the last five years, severe allergic or anaphylactic reaction to rituximab, active viral, bacterial, or fungal infection or history of intravenous antibiotic therapy within four weeks, pregnancy or breastfeeding, acute deterioration of graft function (eGFR decline within 1–3 months > 20%), abnormal hematologic tests (hemoglobin < 7 g/dL, platelet count < 50,000/mm^3^), abnormal liver function tests (alanine aminotransferase, aspartate aminotransferase > 80 IU), rejection classified Banff 2015 criteria grade ≥ I within 1–3 months, psychiatric illness, and alcohol or drug misuse within 1–6 months. To achieve homogeneity within the study population, patients not receiving tacrolimus were also excluded.

Eligible participants who offered consent were randomly assigned to receive rituximab (IVIG–) or rituximab combined with IVIG (IVIG +) at a ratio of 1:1 (stratified by dnDSA peak level: < 10,000 MFI or ≥ 10,000 MFI and participating center). Both groups received rituximab (Roche, Basel, Switzerland) at a dose of 375 mg/m^2^ (maximum 500 mg) on day 0, and the IVIG + group additionally received high-dose IVIG (IV-Globulin®, GC Biopharma, Yongin-si, Republic of Korea; 2 g/kg after rituximab infusion).

### Data collection and follow-up

The clinical and laboratory findings of the patients were assessed at baseline and 3 and 12 months. Assessment of general health status, medication use, and the occurrence of adverse events were performed at every visit. Laboratory measurements were performed at every visit, including DSA, serum creatinine, complete blood count, routine chemistry tests, electrolytes, and urinalysis. The presence of DSA was assessed using LABScreen Single Antigen Class I/Class II (One Lambda, Canoga Park, CA, USA). CD19 levels were measured at baseline and 3 months. The date of the last follow-up was August 2021.

### Study outcome

The primary endpoint was the change in dnDSA titer at 3 and 12 months after preemptive treatment. The dnDSA titer was determined using either strength of immune-dominant class II dnDSA (MFI) or sum of class II dnDSA (MFI). Immune-dominant dnDSA was defined as DSA with the highest MFI at baseline. The sum of class II dnDSA was calculated by adding the MFI values of all class II dnDSAs. The secondary outcomes were changes in eGFR estimated by the Chronic Kidney Disease Epidemiology Collaboration equation^15^ and incident antibody-mediated rejection.

### Statistical analyses

Continuous variables were presented as means with standard deviations (SD) for normally distributed data and medians with interquartile ranges (IQRs) for non-normally distributed data. Categorical variables were expressed as numbers with percentages. Between-group comparisons were tested for statistical significance using the Pearson chi-square test, Fisher exact test, unpaired t-test, Mann–Whitney U test, one-way ANOVA test, or Kruskal–Wallis test, as appropriate. Bonferroni correction was used for P-values to correct for the problem of multiple testing. Generalized estimating equation (GEE) models were used to examine the effect of the treatment on dnDSA titer between the IVIG + and IVIG- groups at 3 and 12 months with baseline. Detailed information about GEE model can be found in the supplementary Material. The MFI values were log-transformed because of their skewed distributions. To examine whether the dnDSA titer change depended on the treatment, the interaction terms between the visit time and treatment group were included in the GEE models. Additionally, both treatment groups (IVIG + and IVIG-) were compared to the no-treatment group. The no-treatment group was defined as patients who had dnDSA but did not receive any treatment (rituximab or IVIG), and these patients were retrospectively recruited from Severance Hospital. The no-treatment group was followed up for 12 months from the onset of dnDSA same as treatment groups. All statistical analyses were performed using R (version 3.5.1; http://www.r-project.org; R Foundation for Statistical Computing, Vienna, Austria), with *p* value < 0.05 considered significant.

## Results

### Baseline characteristics

**Figure 1:**
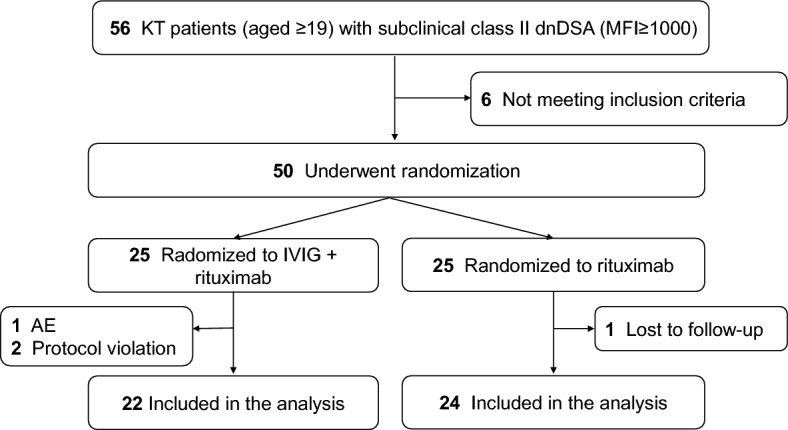
Study flow diagram. KT—kidney transplantation; dnDSA—de novo donor-specific antibody; AE—adverse event; IVIG—intravenous immunoglobulin.

Of the 56 patients initially screened, 50 were randomly assigned in a 1:1 ratio to receive rituximab with IVIG (IVIG+ group) or rituximab alone (IVIG– group). Four patients did not complete the study (adverse event, 1; protocol violation, 2; and follow-up loss, 1). A total of 46 patients (22 in the IVIG+ group and 24 in the IVIG− group) were included in the complete case analyses (Figure 1). The two groups' demographic and baseline laboratory findings were generally similar (Table 1). The median class I and II cPRA% values were 0.0% and 62.8%, respectively. The mean age at enrollment and at kidney transplant were 48.9 and 41.3 years, respectively; most were living-related kidney transplants (73.9%). Most had one class II DSA (80.4%), and DSA against DQ (69.6%) was more common than DSA against DR (30.4%). The median peak MFI of class II dnDSA was 6934.5, and the median MFI sum of class II dnDSA was 7788.5. In both groups, B cells were well depleted, and there was no difference in the number of CD19^+^ B cells at 3 months (*p*=0.669).

**Table 1:** Baseline characteristics.

	Total (N = 46)	IVIG + (IVIG + Rituximab) (n = 22)	IVIG − (Rituximab) (n = 24)	*P*
Age, mean (SD)	48.9 (12.2)	46.1 (12.4)	51.4 (11.7)	0.147
Age at KT, mean (SD)	41.4 (13.5)	38.0 (14.3)	44.5 (12.2)	0.103
Female, n (%)	14 (30.4)	5 (22.7)	9 (37.5)	0.443
Baseline eGFR, mean (SD)	66.7 (16.3)	68.6 (18.4)	65.0 (14.3)	0.465
Urine protein-to-creatinine ratio (g/g)	0.1 [0.1 to 0.3]	0.1 [0.1 to 0.2]	0.1 [0.1 to 0.3]	0.449
*Causes of ESKD, n (%)*				0.206
Glomerulonephritis	13 (28.3)	6 (27.3)	7 (29.2)	
Diabetic nephropathy	7 (15.2)	2 (9.1)	5 (20.8)	
Hypertension	3 (6.5)	1 (4.5)	2 (8.3)	
Other	10 (21.7)	8 (36.4)	2 (8.3)	
Unknown	13 (28.3)	5 (22.7)	8 (33.3)	
*Predialysis, n (%)*				0.113
Hemodialysis	29 (63.0)	11 (50.0)	18 (75.0)	
Peritoneal dialysis	6 (13.0)	5 (22.7)	1 (4.2)	
Preemptive	11 (23.9)	6 (27.3)	5 (20.8)	
Predialysis duration (months), median [IQR]	3.7 [0.3 to 23.4]	5.5 [0.7 to 33.2]	1.6 [0.2 to 20.8]	0.322
*Donor*				0.315
Deceased	3 (6.5)	2 (9.1)	1 (4.2)	
Living-related	34 (73.9)	14 (63.6)	20 (83.3)	
Living-unrelated	9 (19.6)	6 (27.3)	3 (12.5)	
Desensitization, n (%)	4 (8.7)	1 (4.5)	3 (12.5)	0.609
*HLA A mismatch, n (%)*				0.339
0	11 (23.9)	7 (31.8)	4 (16.7)	
1	28 (60.9)	11 (50.0)	17 (70.8)	
2	7 (15.2)	4 (18.2)	3 (12.5)	
*HLA B mismatch, n (%)*				0.185
0	2 (4.3)	2 (9.1)	0 (0.0)	
1	32 (69.6)	13 (59.1)	19 (79.2)	
2	12 (26.1)	7 (31.8)	5 (20.8)	
*HLA DR mismatch, n (%)*				0.536
0	3 (6.5)	2 (9.1)	1 (4.2)	
1	34 (73.9)	17 (77.3)	17 (70.8)	
2	9 (19.6)	3 (13.6)	6 (25.0)	
*HLA DQ mismatch, n (%)*				0.438
0	3 (6.5)	2 (9.1)	1 (4.2)	
1	20 (43.5)	9 (40.9)	11 (45.8)	
2	8 (17.4)	2 (9.1)	6 (25.0)	
Unknown	15 (32.6)	9 (40.9)	6 (25.0)	
Class I cPRA %, median	0.0 [0.0 to 13.0]	0.0 [0.0 to 13.0]	0.0 [0.0 to 13.0]	0.912
*Class I DSA, n (%)*				0.264
0	43 (93.5)	22 (100.0)	21 (87.5)	
1	3 (6.5)	0 (0.0)	3 (12.5)	
Class II cPRA %, median [IQR]	68.0 [48.0 to 81.0]	68.0 [58.0 to 81.0]	58.5 [27.5 to 81.0]	0.159
Class II DSA, MFI sum, median [IQR]	7788.5 [3048.0 to 16,994.0]	9098.0 [3180.0 to 18,407.0]	6977.5 [3024.0 to 14,994.5]	0.439
Class II DSA, MFI peak, median [IQR]	6934.5 [3000.0 to 16,994.0]	6934.5 [3180.0 to 18,407.0]	6977.5 [2657.0 to 14,321.5]	0.478
*Class II immune-dominant DSA*				1.000
DQ	32 (69.6)	15 (68.2)	17 (70.8)	
DR	14 (30.4)	7 (31.8)	7 (29.2)	
*Class II DSA, n (%)*				0.861
1	37 (80.4)	17 (77.3)	20 (83.3)	
2	7 (15.2)	4 (18.2)	3 (12.5)	
3	2 (4.3)	1 (4.5)	1 (4.2)	
CD 19, median [IQR]	11.8 [7.2 to 126.0]	12.6 [6.3 to 143.0]	10.6 [7.2 to 49.2]	0.878
*Immunosuppression*				
Induction therapy				0.718
Yes, n (%)	37 (80.4)	17 (77.3)	20 (83.3)	
Basiliximab, n (%)	37 (80.4)	17 (77.3)	20 (83.3)	
No, n (%)	9 (19.6)	5 (22.7)	4 (16.7)	
*Maintenance therapy*				
Steroids, n (%)	44 (95.7)	20 (90.9)	24 (100.0)	0.432
Tacrolimus, n (%)	46 (100.0)	22 (100.0)	24 (100.0)	1.000
Mycophenolate mofetil, n (%)	35 (76.1)	16 (72.7)	19 (79.2)	0.556
Mizoribine, n (%)	10 (21.7)	6 (27.3)	4 (16.7)	0.608

*IVIG* intravenous immunoglobulin; *SD* standard deviation; *KT* kidney transplant; *eGFR* estimated glomerular filtration rate; *ESKD* end-stage kidney disease; *IQR* interquartile range; *HLA* human leukocyte antigen; *PRA* panel-reactive antibody; *DSA* donor-specific antibody; *MFI* mean fluorescent intensity; *ATG* anti-thymocyte globulin.

### Change in immunodominant dnDSA titer

**Figure 2:**
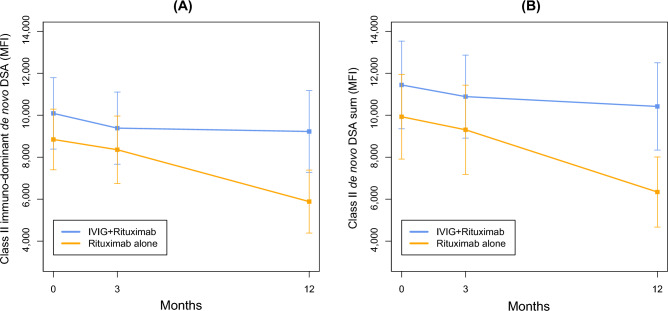
Changes in DSA titer following treatment. (**A**) Serial class II immuno-dominant de novo DSA before and after treatment. Each value are expressed as mean and standard error of the mean. (**B**) Serial class II de novo DSA sum before and after treatment. Each value is expressed as mean and standard error of the mean. DSA—donor-specific antibody; MFI—mean fluorescence intensity; IVIG—intravenous immunoglobulin.

Immune-dominant dnDSA levels decreased at 3 and 12 months after treatment in the IVIG + and IVIG– groups (Fig. 2A). There was no significant difference between the two groups in change of the immune-dominant DSA at 3 and 12 months compared to baseline (Table 2). In the GEE model, there was no difference in immune-dominant class II dnDSA between the two groups (*β* = 0.19; 95% confidence interval [CI], − 0.48 to 0.86; *p* = 0.571, group effect). The immune-dominant class II dnDSA significantly decreased in the IVIG– group at 12 months compared to the baseline (*β* =  − 2.18; 95% CI, − 3.49 to − 0.87; *p* = 0.001, time effect). However, there was no significant difference in immune-dominant class II dnDSA reduction during 12 months between the IVIG- group and the IVIG + group (*β* = 0.91; 95% CI, − 0.82 to 2.63; *p* = 0.303, group*time effect, Table 3). Additionally, anti-DQ DSAs were associated with a lower treatment effect.

**Table 2:** DSA, and renal function at 3 and 12 months.

	3 months		p*	12 months		p*
	IVIG + (n = 22)	IVIG– (n = 24)		IVIG + (n = 22)	IVIG − (n = 24)	
Change in class II immune-dominant dnDSA (MFI), median [IQR]	− 780 [− 2602 to 1014]	− 561 [− 1722 to 149]	1.000	− 1375 [− 2920 to 469]	− 1728 [− 3670 to -943]	0.610
Class in class II dnDSA sum (MFI), median [IQR]	− 780 [− 2602 to 2545]	− 561 [− 1784 to 164]	1.000	− 1207 [− 3605 to 2450]	− 1750 [− 5739 to − 1024]	0.328
eGFR difference, mean (SD)	0.1 (6.3)	− 0.9 (8.6)	1.000	− 3.5 (6.3)	− 2.2 (6.7)	0.988
Urine protein-to-creatinine ratio, g/g, median [IQR]	0.00 [− 0.01 to 0.13]	0.00 [− 0.06 to 0.02]	0.336	0.01 [− 0.01 to 0.09]	0.00 [− 0.02 to 0.04]	1.000

*IVIG* intravenous immunoglobulin; *dnDSA* de novo donor-specific antibody; *MFI* mean fluorescence intensity; *IQR* interquartile range; *cPRA* calculated panel-reactive antibody; *SD* standard deviation; *eGFR* estimated glomerular filtration rate.

Comparison of groups was based on the difference between baseline and each visit time. Group comparisons for normal distribution were performed using the *t* test. Non-normally distributed measurements were compared using the Mann–Whitney *U* test.

*Corrected using Bonferroni’s method for multiple testing.

**Table 3:** Generalized estimating equation analysis for comparing the treatment effect of IVIG/rituximab with that of rituximab alone on class II immuno-dominant dnDSA.

	Group effect		Time effect		Group * time effect		HLA-DQ	
	β (95% CI)	*P*	β (95% CI)	*P*	β (95% CI)	*P*	β (95% CI)	*P*
Baseline	0.19 (− 0.48 to 0.86)	0.571	–	–	–	–	1.93 (0.40 to 3.47)	0.014
3 M			− 0.74 (− 1.56 to 0.08)	0.076	− 0.06 (− 1.34 to 1.21)	0.922		
12 M			− 2.18 (− 3.49 to − 0.87)	0.001	0.91 (− 0.82 to 2.63)	0.303		

*IVIG* intravenous immunoglobulin; *dnDSA* de novo donor-specific antibody; *HLA* human leukocyte antigen; *CI* confidence interval; *MFI* mean fluorescence intensity.

Visit was treated as a categorical variable. The MFI values were log-transformed. β refers to the coefficient estimated from the multivariate generalized estimating equation model. The β coefficients of the group × time interaction terms represent the difference in mean changes in MFI values at each time point with respect to the baseline between the IVIG + and IVIG − groups (mean change in IVIG + group − mean change in IVIG– group).

### Change in the sum of dnDSA titer

The sum of dnDSA also decreased at 3 and 12 months after treatment in the IVIG + and IVIG − groups (Fig. 2B). The sum of class II dnDSA MFI or eGFR changes did not differ between the two groups at either 3 or 12 months (Table 2). In the GEE model, there was no difference in the sum of class II dnDSA MFI between the two groups (*β* = 0.20; 95% CI, − 0.35 to 0.74; *p* = 0.477, group effect). The reduction rate from baseline was significant in the IVIG– group at 12 months (*β* =  − 2.31; 95% CI, − 3.70 to − 0.93; *p* = 0.001, time effect), whereas there was no significant difference in the reduction rate between the IVIG- group and the IVIG + group at 12 months (*β* = 1.41; 95% CI, − 0.26 to 3.07; *p* = 0.098, group*time effect) (Table 4).

**Table 4:** Generalized estimating equation analysis for comparing the treatment effect of IVIG/rituximab with that of rituximab alone on class II dnDSA sum.

	Group effect		Time effect		Group * time effect	
	β (95% CI)	*P*	β (95% CI)	*P*	β (95% CI)	*P*
Baseline	0.20 (− 0.35 to 0.74)	0.477	–	–	–	–
3 M			− 0.75 (− 1.57 to 0.07)	0.073	0.32 (− 0.75 to 1.40)	0.551
12 M			− 2.31 (− 3.70 to − 0.93)	0.001	1.41 (− 0.26 to 3.07)	0.098

*IVIG* intravenous immunoglobulin; *dnDSA* de novo donor-specific antibody; *CI* confidence interval; *MFI* mean fluorescence intensity.

Visit was treated as a categorical variable. The MFI values were log-transformed. β refers to the coefficient estimated from the multivariate generalized estimating equation model. The β coefficients of the group × time interaction terms represent the difference in mean changes in MFI values at each time point with respect to the baseline between the IVIG + and IVIG − groups (mean change in IVIG + group − mean change in IVIG − group).

### Change in kidney function

There was no significant difference in eGFR or proteinuria change between the IVIG + and IVIG– groups at either 3 or 12 months compared to baseline (Table 2). In the GEE model, there was no significant difference in kidney function between the groups at 3 and 12 months (group effect, Table S1). There was also no significant decrease in kidney function in either group at 3 and 12 months (time effect, Table S1).

### Adverse events and antibody-mediated rejection

During the study period, 36 and 41 adverse events occurred in the IVIG + and IVIG − groups, respectively, and there was no difference between the two groups (*p* = 1.000). According to the Spilker classification, adverse events were mild in more than 80% of the patients in both groups. Adverse events were not related to the treatment (61.0% in the IVIG– group), which was higher than that in the IVIG + group (Table S2). No antibody-mediated rejection occurred in either group during the study.

### Comparison between treatment group and no treatment group

To verify the effect of treatment on dnDSA, patients who had subclinical dnDSA but did not receive any treatment (rituximab or IVIG) were retrospectively enrolled as a control group and compared with patients participating in the present study. The two treatment groups' demographic and baseline laboratory findings were generally similar (Table S3). Reduction rate in the titer of class II immune-dominant dnDSA for 12 months was significantly higher in the treatment group (*β* =  − 0.16; 95% CI, − 0.25 to − 0.07; *p* < 0.001, IVIG + and IVIG− groups, group*time effect) compared to the no-treatment group (Table S4). Similarly, the reduction effect of the class II dnDSA sum for 12 months was higher in the treatment group than in the control group (*β* =  − 0.15; 95% CI, − 0.25 to − 0.06; *p* = 0.002, group*time effect, Table S5).

## Discussion

In this open-label randomized controlled trial, we found that preemptive treatment in patients with dnDSA was associated with a reduction in dnDSA MFI. However, compared with the use of rituximab alone, combination of IVIG to rituximab did not provide additional benefit for dnDSA reduction. dnDSA-induced vascular inflammation and microangiopathy play a major role in antibody-mediated rejection, leading to kidney allograft loss^6,16–19^. dnDSA often induced subclinical antibody-mediated rejection as well as overt acute and chronic antibody-mediated rejection^4,5^. For example, over 40% of patients with dnDSA are diagnosed with biopsy-proven subclinical antibody-mediated rejection, leading to progressive graft injury^20^. Subclinical antibody-mediated rejection has also been associated with poor allograft survival, although clinical rejection is associated with worse allograft survival^3,4,21^. However, treatment of subclinical rejection using plasmapheresis, rituximab, and/or IVIG improved graft outcomes to a greater extent than clinical antibody-mediated rejection^14,21^. These results suggest a potential role of early detection and intervention for dnDSA, which can often be combined with subclinical antibody-mediated rejection.

Most previous studies examined the reduction of DSA titer in pre-transplant desensitization of highly sensitized recipients^9,10^ or the treatment of overt rejection using rituximab or rituximab with IVIG^11^. A combination of rituximab and IVIG was used as a desensitization treatment for highly sensitized candidates with preexisting DSA^9^. Desensitization treatment, including rituximab, significantly decreased the incidence of dnDSA 2 years after kidney transplantation^10^. A prospective observational study of patients with dnDSA within 1 year after kidney transplantation and no histological evidence of rejection showed that high-dose IVIG alone was insufficient to reduce MFI to prevent acute antibody-mediated rejection^22^. In contrast, another study on pediatric kidney transplant patients demonstrated that treatment using various combinations of high-dose IVIG, rituximab, and plasmapheresis according to dnDSA strength and biopsy findings significantly reduced class II dnDSA in 11 (73.3%) of 15 patients with subclinical or clinical rejection^23^. However, we excluded plasmapheresis in pre-emptive treatment for subclinical dnDSA, because it needs admission and a relatively long hospital stay compared with convenient therapy of IVIG and rituximab.

No randomized controlled study has investigated the effectiveness of pre-emptive treatment in kidney transplant patients with dnDSA. Therefore, it is not established whether preemptive treatment for dnDSA could be useful or which treatment is better in kidney transplant patients. This randomized clinical trial showed that rituximab alone or combination therapy of rituximab and high-dose IVIG treatment reduced dnDSA MFI. However, our study did not demonstrate clear additional benefits of IVIG combination compared to rituximab alone. As clinical antibody-mediated rejection was not observed in either treatment group during the study period, we could not assess impact of each treatment on the occurrence of antibody-mediated rejection.

Among class II dnDSA, anti-DQ dnDSA showed poorer response to treatment than anti-DR dnDSA. In the previous study, most of the immune-dominant DSAs detected in patients with late rejection were anti-DQ DSA and their titer did not respond well to the rejection treatment^24^. However, the reason why anti-DQ DSA did not respond has not been clearly identified. Further studied to investigate this phenomenon would be needed since anti-DQ dnDSA was associated with poor graft outcome after KT^25–27^.

Considering the poor prognosis of dnDSA and the accompanying antibody-mediated rejection, early detection, and preemptive treatment of dnDSA with or without subclinical rejection could be important. Furthermore, preventive strategies are of course more important. Since class II eplet mismatch is an important immunologic risk factor for dnDSA, good eplet matching can be an important method to reduce the development of DSA^28^. Noncompliance is found in 90% of cases with DSA and clinical rejection and 24% of cases with DSA and subclinical rejection^4^. Therefore, monitoring and preventing non-compliance with immunosuppressants is the most important effort against dnDSA development^29,30^. Immunosuppression-independent risk factors for dnDSA development include younger age, African American, and male^31^. We had better regularly monitor the development of dnDSA, especially in high-risk patients.

This study has several limitations. First, this study enrolled a relatively small number of patients and most of the patients had living-related kidney transplant. Therefore, the potential selection bias could not be ruled out. In addition, the short follow-up duration made it impossible to investigate preemptive treatment's effect on antibody-mediated rejection, kidney function, and graft loss. To overcome this limitation, MFI change was defined as primary endpoint in this study. Based on this study, further large-scale studies with long-term follow-up are needed to assess the long-term benefits of preemptive treatment for dnDSA. Secondly, the lack of allograft biopsy findings at the time of dnDSA detection in this study could not determine the presence of subclinical antibody-mediated rejection. However, the necessity for allograft biopsy in patients with subclinical dnDSAs has not been established in large clinical trials^4^. Third, our additional analysis to compare the preemptive treatment group and the no-treatment group was not a randomized controlled comparison but a preliminary comparison.

Nevertheless, this study is the first, randomized, controlled study to assess effectiveness of preemptive therapy for subclinical dnDSA and determine a better treatment regimen for subclinical dnDSA in kidney transplant patients. This study could contribute to the field by providing baseline data for establishing effective preemptive treatment of subclinical dnDSA.

In conclusion, the preemptive administration of high-dose IVIG combined with rituximab did not show an additional benefit in reducing dnDSA compared to the administration of rituximab alone, although both treatments reduced dnDSA.

## Supplementary Information

Supplementary Information.

## Data Availability

The datasets generated and/or analyzed during the current study are not publicly available due privacy but are available from the corresponding author on reasonable request.

## Abbreviations

DSA: Donor-specific antibody
KT: Kidney transplant
dnDSA: De novo donor-specific antibody
IVIG: Intravenous immunoglobulin
IRB: Institutional review board
eGFR: Estimated glomerular filtration rate
MFI: Mean fluorescent intensity
SD: Standard deviation
IQR: Interquartile ranges
GEE: Generalized estimating equation
