# Supplementary material for: Comparison of high-dose IVIG and rituximab versus rituximab as a preemptive therapy for de novo donor-specific antibodies in kidney transplant patients
Source: Sci Rep. 2023 May 11;13:7682. doi: 10.1038/s41598-023-34804-6 (PMC10175554; doi:10.1038/s41598-023-34804-6)
Supplement: Supplementary file 1 — Supplementary Information. [file 41598_2023_34804_MOESM1_ESM.docx]

**Supplementary Materials**

**Comparison of high-dose IVIG and rituximab versus rituximab as a preemptive therapy for de novo donor-specific antibodies in kidney transplant patients**

**Corresponding author:** Jaeseok Yang (jcyjs@yuhs.ac) and Kyu Ha Huh (KHHUH@yuhs.ac)

**Supplemental Method**

**Table S1.** Generalized estimating equation analysis for comparing the treatment effect of IVIG/rituximab with that of rituximab alone on kidney function

**Table S2.** Adverse events during study period

**Table S3.** Baseline characteristics of patients including historic control

**Table S4.** Generalized estimating equation analysis for the treatment effect on class II immuno-dominant dnDSA (Treat group vs. control group)

**Table S5.** Generalized estimating equation analysis for the treatment effect on class II dnDSA sum (Treat group vs. control group)

**Supplemental Method**

Generalized estimating equation (GEE) models were used to examine the effect of the treatment on de novo donor-specific antibody titer between the rituximab combined with intravenous immunoglobulin (IVIG+) and rituximab (IVIG-) groups at 3 and 12 months with baseline in terms of (1) between-group differences (group effects), (2) within-group changes from baseline (time effects), and (3) between-group differences in change from baseline (group-by-time interaction effects). The β coefficient was estimated using multivariate GEE models. The β coefficients of the group-by-time interaction terms represent the difference in mean changes at each visit concerning the baseline between the IVIG+ and IVIG− groups (mean change in IVIG+ group − mean change in IVIG− group).

**Table S1.** Generalized estimating equation analysis for comparing the treatment effect of IVIG/rituximab with that of rituximab alone on kidney function

|  | **Group effect** | | **Time effect** | | **Group * time effect** | |
| --- | --- | --- | --- | --- | --- | --- |
|  | **β (95% CI)** | ***P*** | **β (95% CI)** | ***P*** | **β (95% CI)** | ***P*** |
| **Baseline** | 0.04 (-0.11 to 0.19) | 0.610 | - | - | - | - |
| **3M** |  |  | -0.02 (-0.07 to 0.03) | 0.450 | 0.01 (-0.05 to 0.07) | 0.720 |
| **12M** |  |  | -0.05 (-0.10 to 0.01) | 0.100 | -0.01 (-0.08 to 0.05) | 0.720 |

***Note:*** Visit was treated as a categorical variable. β refers to the coefficient estimated from the multivariate generalized estimating equation model. The β coefficients of the group × time interaction terms represent the difference in mean changes in MFI values at each time point with respect to the baseline between the IVIG+ and IVIG- groups (mean change in IVIG+ group − mean change in IVIG- group).

***Abbreviations:*** dnDSA, de novo donor-specific antibody; IVIG, intravenous immunoglobulin.

**Table S2.** Adverse events during study period

|  | **IVIG + group** | **IVIG - group** | ***P*** |
| --- | --- | --- | --- |
| **Any adverse events, n** | 36 | 41 |  |
| **Spilker classification** |  |  | 1.000 |
| Mild, n (%) | 29 (80.6) | 34 (82.9) |  |
| Moderate, n (%) | 7 (19.4) | 7 (17.1) |  |
| Severe, n (%) | 0 (0.0) | 0 (0.0) |  |
| **Relationship to study drug** |  |  | 0.004 |
| Not related | 10 (27.8) | 25 (61.0) |  |
| Possible | 11 (30.6) | 11 (26.8) |  |
| Probable | 15 (41.7) | 5 (12.2) |  |

**Table S3.** Baseline characteristics of patients including historic control

|  | **Total**  **(N=77)** | **Control group**  **(n=31)** | **Treat group**  **(n = 46)** | ***P*** |
| --- | --- | --- | --- | --- |
| **Age, mean (SD)** | 50.1 (11.5) | 51.9 (10.4) | 48.9 (12.2) | 0.265 |
| **Female, n (%)** | 45 (58.4) | 31 (100.0) | 14 (30.4) | <0.001 |
| **Baseline eGFR, median [IQR]** | 59.0 [47.7 to 72.1] | 48.0 [37.0 to 55.2] | 66.6 [54.6 to 79.0] | <0.001 |
| **Causes of ESKD, n (%)** |  |  |  | 0.494 |
| Glomerulonephritis | 21 (27.3) | 8 (25.8) | 13 (28.3) |  |
| Diabetic nephropathy | 12 (15.6) | 5 (16.1) | 7 (15.2) |  |
| Hypertension | 3 (3.9) | 0 (0.0) | 3 (6.5) |  |
| Other | 15 (19.5) | 5 (16.1) | 10 (21.7) |  |
| Unknown | 26 (33.8) | 13 (41.9) | 13 (28.3) |  |
| **Predialysis, n (%)** |  |  |  | 0.161 |
| Hemodialysis | 47 (61.0) | 18 (58.1) | 29 (63.0) |  |
| Peritoneal dialysis | 15 (19.5) | 9 (29.0) | 6 (13.0) |  |
| Preemptive | 15 (19.5) | 4 (12.9) | 11 (23.9) |  |
| **Predialysis duration (months), median [IQR]** | 8.0 [1.2 to 32.0] | 14.0 [3.0 to 51.0] | 3.7 [0.3 to 23.4] | 0.100 |
| **Donor** |  |  |  | <0.001 |
| Deceased | 10 (13.0) | 7 (22.6) | 3 (6.5) |  |
| Living-related | 43 (55.8) | 9 (29.0) | 34 (73.9) |  |
| Living-unrelated | 24 (31.2) | 15 (48.4) | 9 (19.6) |  |
| **Desensitization** |  |  |  | 0.012 |
| No | 61 (79.2) | 20 (64.5) | 41 (89.1) |  |
| Yes | 15 (19.5) | 11 (35.5) | 4 (8.7) |  |
| Unknown | 1 (1.3) | 0 (0.0) | 1 (2.2) |  |
| **HLA A mismatch, n (%)** |  |  |  | 0.893 |
| 0 | 18 (23.4) | 7 (22.6) | 11 (23.9) |  |
| 1 | 46 (59.7) | 18 (58.1) | 28 (60.9) |  |
| 2 | 13 (16.9) | 6 (19.4) | 7 (15.2) |  |
| **HLA B mismatch, n (%)** |  |  |  | 0.137 |
| 0 | 2 (2.6) | 0 (0.0) | 2 (4.3) |  |
| 1 | 49 (63.6) | 17 (54.8) | 32 (69.6) |  |
| 2 | 26 (33.8) | 14 (45.2) | 12 (26.1) |  |
| **HLA DR mismatch, n (%)** |  |  |  | 0.053 |
| 0 | 4 (5.2) | 1 (3.2) | 3 (6.5) |  |
| 1 | 50 (64.9) | 16 (51.6) | 34 (73.9) |  |
| 2 | 23 (29.9) | 14 (45.2) | 9 (19.6) |  |
| **HLA DQ mismatch, n (%)** |  |  |  | 0.378 |
| 0 | 4 (5.2) | 1 (3.2) | 3 (6.5) |  |
| 1 | 29 (37.7) | 9 (29.0) | 20 (43.5) |  |
| 2 | 13 (16.9) | 5 (16.1) | 8 (17.4) |  |
| Unknown | 31 (40.3) | 16 (51.6) | 15 (32.6) |  |
| **Class I cPRA %, median** | 0.0 [0.0 to 12.0] | 0.0 [0.0 to 6.5] | 0.0 [0.0 to 13.0] | 0.563 |
| **Class I DSA, n (%)** |  |  |  | 0.406 |
| 0 | 70 (90.9) | 27 (87.1) | 43 (93.5) |  |
| 1 | 6 (7.8) | 3 (9.7) | 3 (6.5) |  |
| 2 | 1 (1.3) | 1 (3.2) | 0 (0.0) |  |
| **Class II cPRA %, median [IQR]** | 68.0 [56.0 to 81.0] | 68.0 [60.0 to 90.0] | 68.0 [48.0 to 81.0] | 0.194 |
| **Class II DSA, MFI sum, median [IQR]** | 9460.0 [3000.0 to 18832.0] | 10431.0 [2711.0 to 21069.5] | 7788.5 [3048.0 to 16994.0] | 0.388 |
| **Class II DSA, MFI peak, median [IQR]** | 7943.0 [2715.0 to 14386.0] | 8498.0 [2711.0 to 13575.5] | 6934.5 [3000.0 to 16994.0] | 0.729 |
| **Class II immune-dominant DSA** |  |  |  | 1.000 |
| DQ | 53 (68.8) | 21 (67.7) | 32 (69.6) |  |
| DR | 24 (31.2) | 10 (32.3) | 14 (30.4) |  |
| **Class II DSA, n (%)** |  |  |  | 0.023 |
| 1 | 53 (68.8) | 16 (51.6) | 37 (80.4) |  |
| 2 | 15 (19.5) | 8 (25.8) | 7 (15.2) |  |
| 3 | 6 (7.8) | 4 (12.9) | 2 (4.3) |  |
| 5 | 3 (3.9) | 3 (9.7) | 0 (0.0) |  |
| **Immunosuppression** |  |  |  |  |
| **Induction** |  |  |  | <0.001 |
| ATG, n (%) | 10 (13.0) | 10 (32.3) | 0 (0.0) |  |
| Basiliximab, n (%) | 56 (72.7) | 19 (61.3) | 37 (80.4) |  |
| No induction, n (%) | 11 (14.3) | 2 (6.5) | 9 (19.6) |  |
| **Maintenance Therapy** |  |  |  |  |
| Steroids, n (%) | 73 (94.8) | 29 (93.5) | 44 (95.7) | 1.000 |
| Tacrolimus, n (%) | 77 (100.0) | 31 (100.0) | 46 (100.0) | - |
| Mycophenolate mofetil, n (%) | 55 (71.4) | 20 (64.5) | 35 (76.1) | 0.316 |
| Mizoribine, n (%) | 17 (22.1) | 6 (19.4) | 11 (23.9) | 0.847 |

***Abbreviations:*** IVIG, intravenous immunoglobulin; eGFR, estimated glomerular filtration rate; ESKD, end-stage kidney disease; IQR, interquartile range; HLA, human leukocyte antigen; PRA, panel-reactive antibody; DSA, donor-specific antibody; MFI, mean fluorescence intensity; ATG, anti-thymocyte globulin.

**Table S4.** Generalized estimating equation analysis for the treatment effect on class II immuno-dominant dnDSA (treat group vs. control group)

|  | **Group effect** | | **Time effect** | | **Group * time effect** | | **HLA-DQ** | |
| --- | --- | --- | --- | --- | --- | --- | --- | --- |
|  | **β (95% CI)** | ***P*** | **β (95% CI)** | ***P*** | **β (95% CI)** | ***P*** | **β (95% CI)** | ***P*** |
| **Visit** | -0.03 (-0.51 to 0.45) | 0.904 | 0.02 (-0.04 to 0.07) | 0.531 | -0.16 (-0.25 to -0.07) | <0.001 | 1.50 (0.44 to 2.56) | 0.005 |

***Note:*** Visit was treated as continuous variables (months). MFI values were log transformed. β refers to the coefficient estimated from the multivariate generalized estimating equations models. The β coefficients of the group × time interaction terms represent the difference in mean changes in MFI values at each time point with respect to the baseline between groups (mean change in treatment group − mean change in control group).

***Abbreviations:*** dnDSA, de novo donor-specific antibody; IVIG, intravenous immunoglobulin; MFI, mean fluorescence intensity.

**Table S5.** Generalized estimating equation analysis for the treatment effect on class II dnDSA sum (Treat group vs. control group)

|  | **Group effect** | | **Time effect** | | **Group * time effect** | |
| --- | --- | --- | --- | --- | --- | --- |
|  | **β (95% CI)** | ***P*** | **β (95% CI)** | ***P*** | **β (95% CI)** | ***P*** |
| **Visit** | -0.15 (-0.72 to 0.42) | 0.604 | 0.02 (-0.04 to 0.08) | 0.512 | -0.15 (-0.25 to -0.06) | 0.002 |

***Note:*** Visit was treated as continuous variables (months). MFI values were log transformed. β refers to the coefficient estimated from the multivariate generalized estimating equations models. The β coefficients of the group × time interaction terms represent the difference in mean changes in MFI values at each time point with respect to the baseline between the groups (mean change in treatment group − mean change in control group).

***Abbreviations:*** dnDSA, de novo donor-specific antibody; IVIG, intravenous immunoglobulin; MFI, mean fluorescence intensity.
